# Rapid and Stable Plasma Transformation of Polyester Fabrics for Highly Efficient Oil–Water Separation

**DOI:** 10.1002/gch2.201900095

**Published:** 2020-03-03

**Authors:** Ye Sun, Bo Ouyang, Rajdeep Singh Rawat, Zhong Chen

**Affiliations:** ^1^ School of Material Science and Engineering Nanyang Technological University Singapore 639798 Singapore; ^2^ Natural Science and Science Education National Institute of Education Nanyang Technological University Singapore 637616 Singapore

**Keywords:** green chemistry, oil cleanups, oil–water separation, polyester

## Abstract

Fabrics with special wettability have drawn growing attention in recent years in the area of oil–water separation due to their low cost, good flexibility, and ease of handling. However, an efficient and fast method to enable the required wetting state on fabrics still remains a challenge. In this work, a one‐step, rapid, and chemical‐free hydrogen plasma treatment is reported to prepare a superhydrophobic and oleophilic polyester fabric. The as‐prepared fabrics display a static water contact angle of 153.2° with excellent oil–water separation capability. The mechanism of surface transformation is discussed through chemical analyses, which indicate a significant removal of carboxyl group from the pristine hydrophilic surface. This developed method is envisaged to be used for on‐demand large‐scale production of materials for emergency oil cleanup through either separation or selective adsorption.

## Graphical abstract

A one‐step, rapid, and chemical‐free method has been developed to prepare a superhydrophobic and oleophilic polyester fabric. The as‐prepared fabrics display great water repellency and excellent oil–water separation capability. This developed method is envisaged to be used for on‐demand large‐scale production of materials for emergency oil cleanup through either separation or selective adsorption.

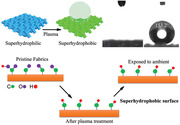

Inspired by nature^[^ ^1^, ^2^ ^]^ super‐hydrophobicity (water contact angle > 150°) has attracted tremendous attention because of its great potential in self‐cleaning and oil–water separation. Mimicking the lotus leaf, superhydrophobic surface can be constructed through micro/nanostructurization of surface, and chemically modifying the surface with low surface energy materials.^[^ ^3^ ^]^ Good progress has been made in developing surperhydrophobic surfaces on various substrate materials including silicon,^[^ ^4^ ^]^ glass,^[^ ^5^, ^6^, ^7^ ^]^ and fabric^[^ ^3^, ^8^, ^9^, ^10^ ^]^ Among them, fabric materials possess a vast potential for practical applications in separation of oil–water mixture or selective oil adsorption owing to their inexpensiveness and good mechanical flexibility.^[^ ^11^, ^12^ ^]^ A pristine fabric absorbs both water and oil when its surface is modified to simultaneously become superhydrophobic and oleophilic. It can be used to selectively absorb oil to separate it from its mixture with water based on the special wetting properties. It is generally more cost‐effective to use fabrics in separation of oil‐water mixture than using other options such as metallic meshes, since extra step of creating surface rough features is required. Additionally, poor corrosion resistance of metallic meshes is poses challenge to their long‐term use.

Numerous methods of fabricating such superhydrophobic fabrics have been investigated including wet‐chemical approaches and dry‐physical process to introduce surface roughness and chemistry.^[^ ^13^ ^]^ So far, most of the existing processes on modifying surface involve coating of low surface energy materials^[^ ^12^ ^]^ such as modifying inorganic nanoparticles SiO_2_,^[^ ^14^, ^15^ ^]^ TiO_2_,^[^ ^16^ ^]^ or using fluorocarbon silane^[^ ^17^ ^]^ on a pre‐roughened surface. These multi‐step processes can be time‐consuming^[^ ^18^ ^]^ and may produce chemical waste. It is of great interest to use a simpler physical process such as ion beam^[^ ^19^ ^]^ or plasma technique to obtain the same effect in a single step with much reduced processing time. We can envisage that such capability will be useful for emergencies when materials have to be supplied quickly and on an on‐demand basis. Plasma treatment can be used to induce either hydrophilicity or hydrophobicity depending on the gas (es) used. For example, Karahan et al. used atmospheric plasma to improve hydrophilicity of cotton fabric to replace the conventional wet process.^[^ ^20^ ^]^ Similarly, superhydrophobic fabric has been prepared in a two‐step process involving plasma treatment to roughen the surface first and grafting a low surface energy material.^[^ ^21^ ^]^ However, a simple one‐step procedure to achieve the superhydrophobicity still remains a challenge. On the other hand, the ability of plasma to modify the surface chemistry and even physical features offers the possibility to achieve the required surface chemistry and/or surface morphology towards the required wettability state in one simple treatment.

Here, we report a rapid, chemical‐free and one‐step process to realize superhydrophobic polyester fabrics (SPFs) via hydrogen plasma treatment. Excellent water contact angle (WCA) of 153.2° has been achieved after 4 min treatment. Such SPFs exhibit excellent separation efficiency of oil–water mixture. The mechanism of transforming originally hydrophilic surface to the superhydrophobic state is elucidated. The strategy provides a novel pathway towards practical applications such as treatment of oil contamination.

The SPF preparation is schematically shown in **Figure** **1**A. Figures 1B and 1C are the water contact images on untreated and treated polyester fabrics (4 min@250 W), respectively. The contact angle of the polyester fabric surface reached 153.2° and water shedding angle (WSA) of 12 ± 1.5° after the plasma treatment, while water droplet was quickly absorbed and completely spread out from the surface of pristine fabric. The excellent flexibility and appearance of the polyester fabrics were well preserved after the plasma treatment.

**Figure 1:**
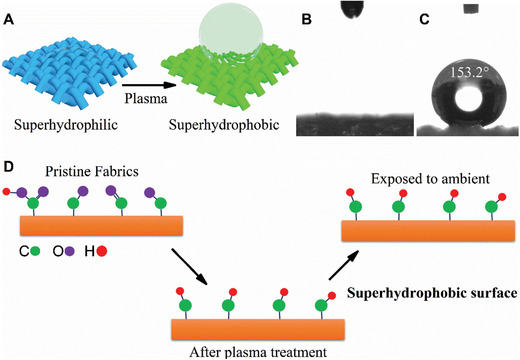
A) Schematic diagram of polyester fabrics, from superhydrophilic to superhydrophobic after plasma treatment; B) untreated fabrics absorb water completely (superhydrophilic); C) as‐prepared superhydrophobic fabrics with WCA above 150° after 4 min of hydrogen plasma treatment at 250 W; and D) schematic of mechanism of surface transformation to superhydrophobic.

To examine the stability of the plasma treated fabrics, the 4 min@250 W SPFs were placed in ambience with humidity of 60–70% for up to eight months. The WCA of fabrics was very stable (**Figure** **2**A,C). In addition, the WCA on treated fabrics was carried out using droplets of pH = 1 (HCl solution) and pH = 14 (NaOH solution). As exhibited in Figure 2B, droplets of HCl and NaOH solution remained spherical and stably stayed on the surface for 1 hr. These results demonstrate that the fabricated SPFs via plasma technique are highly stable. Besides chemical stability, the mechanical properties of the polyester fabric before and after the plasma treatment were compared. Figure 2D displays representative stress–strain curves. The plasma‐treated fabrics have a slight decrease in the mechanical strength (from 26.92 ± 2.20 to 20.13 ± 0.76 MPa) and elastic modulus (from 44.85 ± 0.03 to 35.41 ± 0.02 MPa, as shown in Figure 2E). Such decrease is attributed to the change of chemical bonding state on the surface by the plasma treatment. Nevertheless, the slightly compromised mechanical properties will not affect our intended applications.

**Figure 2:**
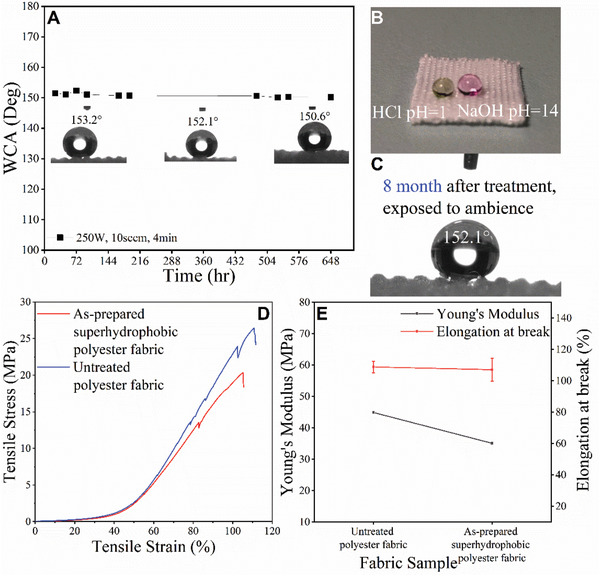
A) WCA measurement of as‐prepared superhydrophobic surface sample versus storage time (h), B) droplets of HCl and NaOH stably stay on plasma‐treated fabric surface, C) after eight‐month storage, D) representative stress–strain curves of untreated and as‐prepared samples; E) Young's Modulus (MPa) and maximum percentage elongation at break point of untreated and as‐prepared samples.

Surface superhydrophobicity is typically derived from combined rough surface and surface chemistry affinity change against water. However, as observed under FESEM, the surface morphology of individual fibers in the SPF has no significant change after the plasma treatment as shown in **Figure** **3**A–C and D–F. Therefore, the only change on the fabric surface comes from the surface chemistry modification, as illustrated in Figure 1D. The fabric itself possesses a micro‐scale rough structure owing to the individual fibers.

**Figure 3:**
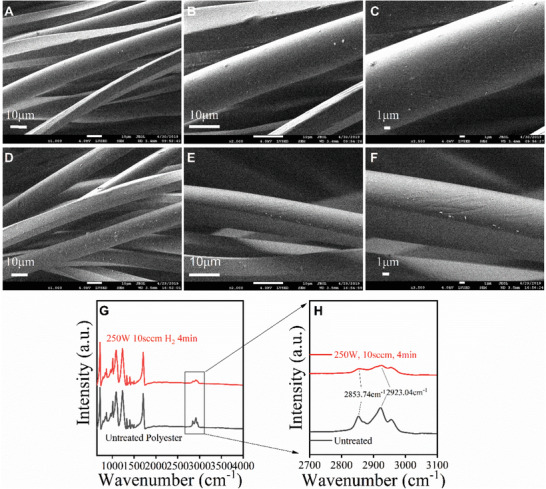
FESEM images of A–C) untreated sample surface and D–F) as‐prepared superhydrophobic polyester fabric surface at different magnification; G, H) FTIR spectra of pristine fabrics and plasma‐treated fabrics.

FTIR spectra of hydrogen RF‐plasma treated polyester and untreated polyester are shown in Figure 3G. There are apparent differences in the vibration peaks between 2800 and 3000 cm^−1^ (Figure 3H) which are related to the carboxyl O—H group stretching. The reduction of carboxylic acid O—H groups peaks is responsible for the increased water resistance of SPF surface after the treatment. Further analysis through XPS was conducted to understand the surface chemical composition changes. As shown in **Figure** **4**A,D, the survey XPS spectra indicate that the polyester fabric before and after plasma treatment was composed of elements C and O only, and no other elements were identified. A considerable reduction of oxygen peak intensity was observed after the plasma treatment, indicating strong oxygen removal (**Table** **1**).

**Figure 4:**
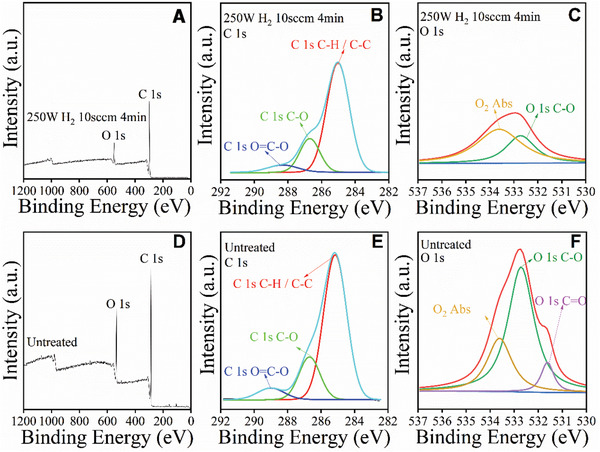
Survey XPS spectra of polyester fabrics: A) 4min@250W hydrogen RF‐plasma treatment at 10sccm and D) untreated polyester; B,C,E,F) High‐resolution fitted XPS spectra of C 1s and O 1s of treated and untreated polyester fabric, respectively.

**Table 1:** Surface composition of C and O before and after the plasma treatment

Untreated		4 min@250 W, H_2_ 10sccm	
C 1s	O 1s	C 1s	O 1s
65.6 at%	34.4 at%	78.4 at%	21.6 at%

Figure 4B and 4E show the C1s core‐level spectra of polyester farbic deconvoluted into binding energy peaks at 288.8, 286.7, and 285.1 eV, which are characterized as C=O, C—O and C—C/C—H, respectively. The O1s core‐level spectra (Figure 4C,F), are fitted into C=O, C=O and Abs‐O_2_, respectively, with binding energy at 531.6, 532.7, and 533.6 eV. Significant reduction of O bonding (C—O and C=O) was detected. The disappearance of C=O based on O 1s spectra (Figure 4C) for plasma treated fabric confirms the strong O‐removal capability of H‐plasma. This is consistent with the FTIR observation that there was an apparent reduction of carboxyl group after the plasma treatment. Our results indicate that the mechanism for transformation of the superhydrophilic surface to superhydrophilic is due to the significant reduction of the surface carboxyl group after the plasma treatment. Correspondingly, the surface energy was substantially reduced.

**Figure** **5**A,B show distinct difference when a piece of the plasma‐treated fabric was placed on oil (Hexane, dyed with oil red for easy observation) and water (dyed with methylene blue). The treated fabric shows oleophilicity (quickly absorbing the oil upon in contact, Figure 5A) and hydrophobicity (floating on water, Figure 5B). Selective wetting was further proven after they were dipped into water and oil (Figure 5C): the as‐prepared SPF repels water but fully absorbs the oil.

**Figure 5:**
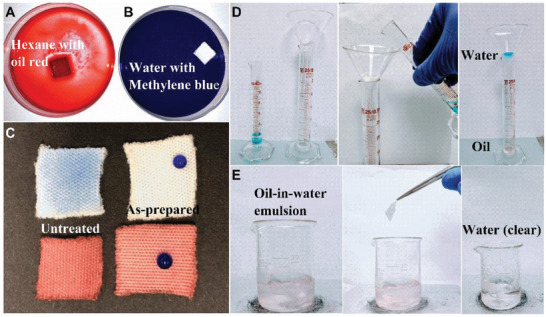
A,B) As‐prepared superhydrophobic polyester fabric absorbs hexane while repels water (floating on water), C) comparison between untreated and as‐prepared superhydrophobic fabrics after dipping in water and oil. Left column: untreated; right column: treated at 4 min@250 W, D) oil‐water mixture separation process with the superhydrophobic fabrics, and E) selective collection of oil with stirring from surfactants free oil in water emulsions.

The treated fabric was used for gravity‐driven separation of oil‐water mixture. The fabric was placed at the nozzle of the funnel. Oil was able to pass through the porous fabric but water could not (Figure 5D). We calculated the oil separation efficiency for 10 cycles using *e* = (*V*−*V_0_*)/*V*, where *V* is the initial volume of oil and *V_0_* the volume of oil lost during separation.^[^ ^22^ ^]^ The superhydrophobic fabrics demonstrated a separation efficiency greater than 90% (up to 92%) for ten cycles of filtration (the lost was due to the adsorption to the fabric).

Furthermore, the as‐prepared SPFs were examined for oil collection from oil‐in‐water surfactant‐free emulsion (Figure 5E). The emulsion was obtained by mixing water and hexane (dyed with oil red) in a volume ratio of 99:1, and then going through intensive stirring for 2 h.^[^ ^23^ ^]^ The fabric was gradually dyed to red color and the remaining mixture slowly became transparent. This suggests successful removal of oil from emulsion through adsorption. We have also measured the amount of oils that can be adsorbed by a unit weight of the treated fabric, the adsorption capacity for n‐Hexane, n‐Hexadecane, Toluene, silicone oil, and soybean oil is 1.5, 2.5, 2.6, 2.9, and 3.5 g per g, respectively.

Our Method to produce superhydrophobic and oleophilic fabrics is highly efficient and chemical‐free. The fabrics are highly stable after exposure in ambient condition for more than eight months. Hydrogen plasma treatment has significantly reduced the surface oxygen containing groups (C—O, C=O), and resulted in reduction of affinity to water. The as‐prepared superhydrophobic polyester fabrics exhibited excellent performance in oil–water separation via filtration and oil collection from emulsion. The reported method has advantages of being rapid, chemical‐free and easy to prepare, paving way for on‐demand production of superhydrophobic fabrics for environmental cleanup and other applications.

## Experimental Section

The SPFs were prepared via a radio frequency (RF) plasma system.^[^ ^24^ ^]^ In a typical procedure, pristine polyester fabric sample (1.5 cm × 1.5 cm) was washed with ethanol and deionized water repeatedly, before being dried at 65 °C. The sample was then placed inside the RF‐plasma system made of a quartz tube between the two capacitively coupled electrodes. Afterwards, the tube was evacuated to 10–3 mbar. Hydrogen gas then was introduced into the system at a flow rate of 10 sccm. The system stabilized at pressure of 0.45 mbar. H2 plasma was created using 250 W RF power from a 13.56 MHz Caeser136 RF generator connected to the ring electrode through auto‐impedance matching unit. The plasma treatment of the polyester fabrics was carried out for 4 min.

The surface features of SPFs were observed using a JEOL 7800F prime field emission scanning microscope (FESEM) operated at 4 kV. A Perkin Elmer Frontier Fourier transformation Infrared (FTIR) spectrometer was used to understand surface molecular changes of the polyester fabric before and after the plasma treatment. Kratos AXIS Supra X‐ray photoelectron spectrometer (XPS) was applied for the surface chemical composition measurements. The binding energy of the C 1s peak from sp2‐bonded carbon at 285 eV was used as a reference. WCA was recorded using a contact angle goniometer (OCA 20 Dataphysics) with 5 µL DI water droplet.

An MTS Criterion (Model 43) tester was used to measure mechanical properties. Five treated samples were measured and compared with the untreated samples under ambient condition. Dog bone shaped samples of 25 mm length along the axis of ward threads and 4 mm width were prepared for the mechanical test. Samples were mounted via the pneumatic clamps and the extension rate was fixed at 5 mm min^−1^. Young's modulus (MPa) and percent elongation were recorded. For each type of sample, an average of five measurements with standard deviations was reported.

## Conflict of Interest

The authors declare no conflict of interest.

## Supporting information

Supporting Information
