# Supplementary material for: Rapid and Stable Plasma Transformation of Polyester Fabrics for Highly Efficient Oil–Water Separation
Source: Glob Chall. 2020 Mar 3;4(7):1900095. doi: 10.1002/gch2.201900095 (PMC7330499; doi:10.1002/gch2.201900095)
Supplement: Supplementary file 1 — Supporting Information [file GCH2-4-1900095-s001.pdf]

Copyright WILEY-VCH Verlag GmbH & Co. KGaA, 69469 Weinheim, Germany, 2020.

Global Challenges
Open Access

Supporting Information

for *Global Challenges,* DOI: 10.1002/gch2.201900095

Rapid and Stable Plasma Transformation of Polyester Fabrics for Highly Efficient Oil–Water Separation

Ye Sun, Bo Ouyang, Rajdeep Singh Rawat, and Zhong Chen**

Supporting Information

Rapid and stable plasma transformation of polyester fabrics for highly efficient oil-water separation

Ye Sun[1], Bo Ouyang[2], Rajdeep Singh Rawat[2,], Zhong Chen[1,]**

Ye Sun, Prof Zhong Chen

School of Material Science and Engineering, Nanyang Technological University, Singapore 639798

Email: ASZChen@ntu.edu.sg

Bo Ouyang, Prof Rajdeep Singh Rawat

Natural Science and Science Education, National Institute of Education, Nanyang Technological University, Singapore 637616

Email: rajdeep.rawat@nie.edu.sg

Effect of hydrogen gas flow rate, RF plasma power and treatment time

To understand the effect of plasma power and hydrogen gas flow on the hydrophobicity of the polyester surface, three different RF powers (50, 250, and 500 W) at different hydrogen gas flow rate (1, 5, and 10 sccm) were used to treat the polyester fabrics for a fixed treatment time of 4 min. Best performance in terms of WCA was observed at plasma power of 250 W as shown in **Figure S1A**, with the WCA above 150°. With a lower power at 50 W, the WCA was around 140°. At the higher plasma power 500W, WCA was slightly lower than 150° (~147°). As shown in high resolution XPS O1s spectra in **Figure S2B**, compared with untreated polyester surface, oxygen bonding is reduced significantly especially at plasma power at 250 W, the binding energy for C=O bonding (peaked at 531.6eV) was not present. While at

50 W, there was less reduction of the oxygen bonding peaks, which is the reason that superhydrophobicity was not achieved at plasma power of 50 W.

Highest WCA was observed at 10 sccm gas flow rate for fixed RF-power of 250 W as shown in **Figure S1B**. The high resolution XPS O1s fitted spectra indicate that at this flow rate, oxygen bonding reduction was the highest among all gas flow rates studied. The best RF-power and flow rate combination was then used for different plasma treatment time of 1, 2, and 4 min. Effect of treatment time is shown in **Figure S1C**. Longer treatment time at 4 min resulted in best WCA performance at 250 W plasma power and 10 sccm gas flow rate. Longer treatment time (more than 4 min) resulted in similar result as 4 min treatment, therefore it is unnecessary to further increase the treatment time, as unnecessarily longer plasma treatment may cause over heating of the sample leading to damage of the heat-sensitive fabrics.

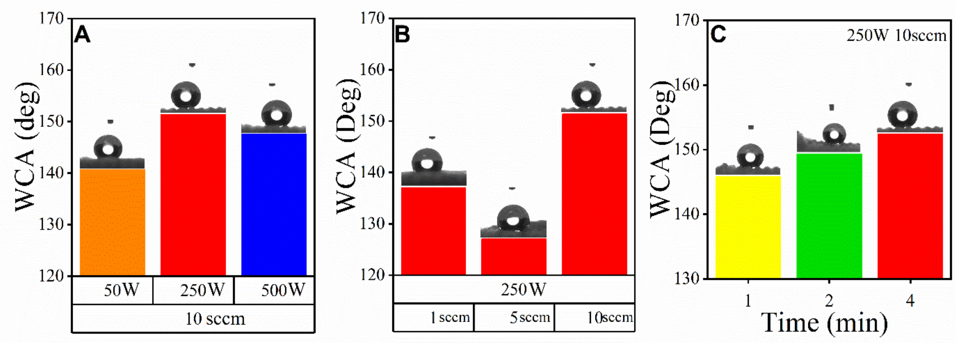

Figure S1. (**A**) WCA under different plasma power with gas flow rate at 10 sccm; (**B**) WCA under different gas flow rate at 250 W plasma power; (**C**) effect of treatment time.

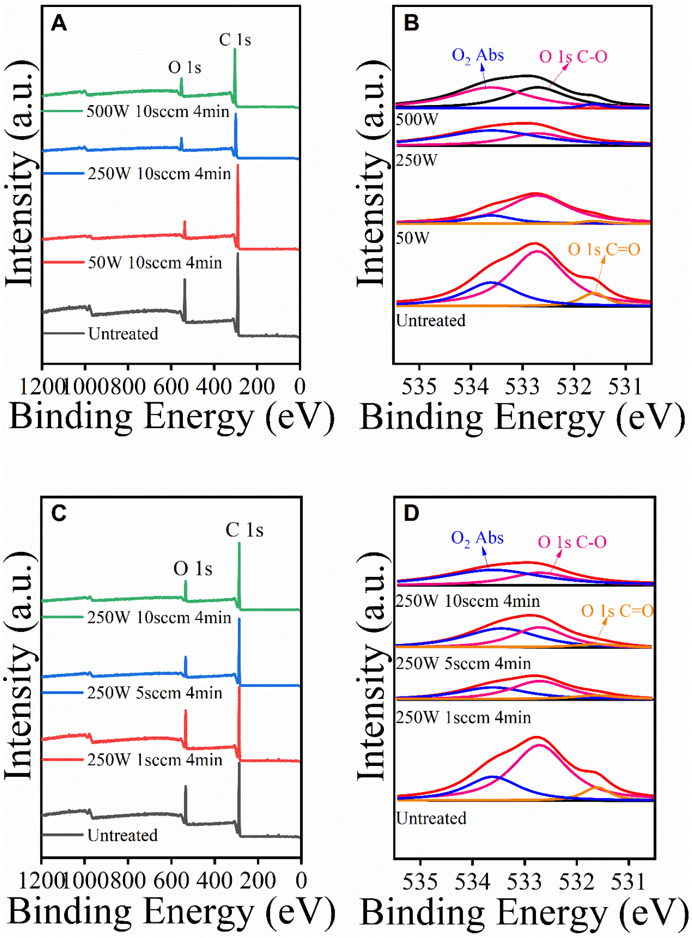

Figure S2. (a) and (c) survey spectrum of surface treated under different plasma power and gas flow rate; (b) and (d) high resolution O1s fitted peak comparison between different plasma power and gas flow rate.
